# Characterization of the *in vitro, ex vivo*, and *in vivo* Efficacy of the Antimicrobial Peptide DPK-060 Used for Topical Treatment

**DOI:** 10.3389/fcimb.2019.00174

**Published:** 2019-05-28

**Authors:** Joakim Håkansson, Lovisa Ringstad, Anita Umerska, Jenny Johansson, Therese Andersson, Lukas Boge, René T. Rozenbaum, Prashant K. Sharma, Petter Tollbäck, Camilla Björn, Patrick Saulnier, Margit Mahlapuu

**Affiliations:** ^1^Division of Bioscience and Materials, RISE Research Institutes of Sweden, Borås, Sweden; ^2^Université de Lorraine, CITHEFOR, Nancy, France; ^3^INSERM 1066, CNRS 6021, Université Bretagne Loire, MINT, UNIV Angers, Angers, France; ^4^Department of Biomedical Engineering, University of Groningen, University Medical Center Groningen, Groningen, Netherlands; ^5^Promore Pharma AB, Solna, Sweden

**Keywords:** antimicrobial peptides, DPK-060, skin infections, lipid nanocapsules, cubosomes

## Abstract

Antimicrobial peptides, also known as host defense peptides, have recently emerged as a promising new category of therapeutic agents for the treatment of infectious diseases. This study evaluated the preclinical *in vitro, ex vivo*, and *in vivo* antimicrobial activity, as well as the potential to cause skin irritation, of human kininogen-derived antimicrobial peptide DPK-060 in different formulations designed for topical delivery. We found that DPK-060 formulated in acetate buffer or poloxamer gel caused a marked reduction of bacterial counts of *Staphylococcus aureus in vitro* (minimum microbicidal concentration <5 μg/ml). We also found that DPK-060 in poloxamer gel significantly suppressed microbial survival in an *ex vivo* wound infection model using pig skin and in an *in vivo* mouse model of surgical site infection (≥99 or ≥94% reduction in bacterial counts was achieved with 1% DPK-060 at 4 h post-treatment, respectively). Encapsulation of DPK-060 in different types of lipid nanocapsules or cubosomes did not improve the bactericidal potential of the peptide under the applied test conditions. No reduction in cell viability was observed in response to administration of DPK-060 in any of the formulations tested. In conclusion, the present study confirms that DPK-060 has the potential to be an effective and safe drug candidate for the topical treatment of microbial infections; however, adsorption of the peptide to nanocarriers failed to show any additional benefits.

## Introduction

The rapidly increasing resistance toward conventional antibiotics has accelerated research efforts to identify new and non-conventional anti-infective therapies (Czaplewski et al., 2016). One category of the recently emerged novel drug candidates for the treatment of infectious disease are antimicrobial peptides (AMPs), also known as host defense peptides (Mahlapuu et al., 2016). AMPs are low mass and generally positively charged peptides, which display a large structural and functional diversity. Most AMPs have the ability to kill microbial pathogens directly, whereas others act indirectly via immunomodulatory actions (Fjell et al., 2011). Importantly, AMPs are generally considered to be less prone to microbial resistance compared with conventional antibiotics (Andersson et al., 2016). A number of AMPs have already been introduced into the market, and additional AMPs are currently being tested in clinical trials (Fox, 2013). In spite of these encouraging examples, there is still a considerable discrepancy between the extensive range of AMPs claimed as potent drug candidates in the patents or related scientific literature and the relatively few reported outcomes of the clinical trials (Kosikowska and Lesner, 2016).

The implementation of innovative formulation strategies is one of the factors, which is expected to accelerate the translation of preclinical candidate AMPs into successful clinical products. Notably, the use of nanocarriers for the delivery of AMPs has recently emerged as one area of interest, since nanoparticles provide unique advantages due to their large surface area for adsorption/encapsulation of AMPs and prevention of self-aggregation of the peptides (Eckert, 2011; Sandreschi et al., 2016; Nordström and Malmsten, 2017).

One of the AMPs, where intense preclinical and clinical research has been conducted in the past, is DPK-060 (also known as GKH17-WWW), which was developed for the treatment of skin infections. DPK-060 is a chemically synthesized peptide structurally derived from human protein kininogen, where three tryptophan residues have been added to the C-terminal end of the endogenous 17-amino acid sequence (Schmidtchen et al., 2009). In comparison to its endogenous analog, this addition enhances the ability of DPK-060 to withstand enzymatic degradation by infection-affiliated proteases, without any signs of aggravated cytotoxicity (Schmidtchen et al., 2009). DPK-060 exhibits a potent broad-spectrum antimicrobial activity *in vitro* against both Gram-positive and Gram-negative bacteria including methicillin-resistant *Staphylococcus aureus* (MRSA; Boge et al., 2017; Nordström et al., 2018). The safety and efficacy of 1% DPK-060 in a polyethylene glycol (PEG)-based ointment has been studied in a clinical phase II trial in the treatment of skin infections in atopic dermatitis patients. The results of this trial revealed that DPK-060 1% ointment was well tolerated by the subjects and significantly reduced the microbial density in eczematous lesions after 14 days with twice daily application compared with placebo (ClinicalTrials.gov Identifier: NCT01522391; EudraCT: 2007-007103-32).

**Figure 1:**
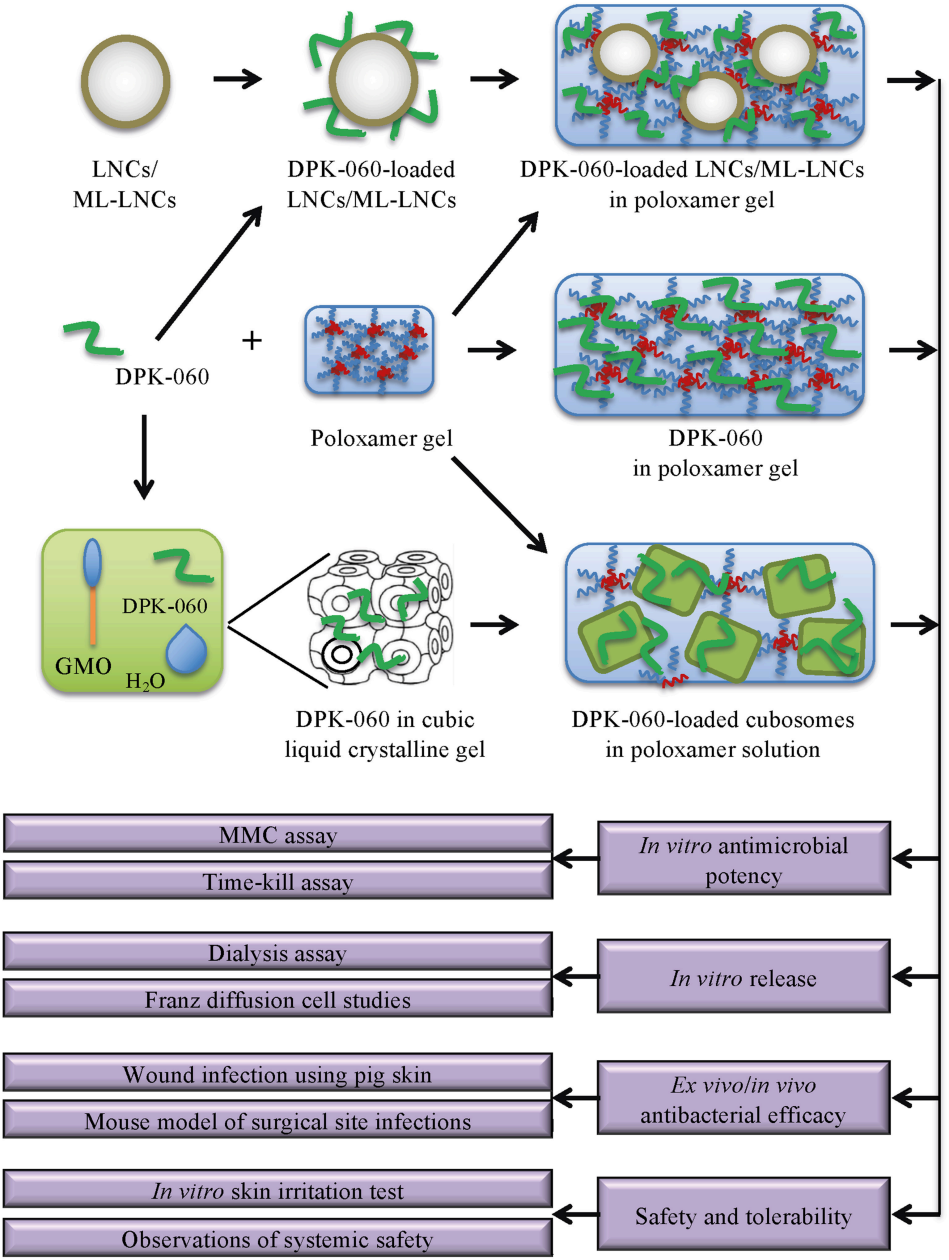
Schematic presentation of the experimental design of the study. LNC/ML-LNC particles were constructed and DPK-060 was adsorbed to the particle surface; DPK060-loaded LNCs/ML-LNCs were then added to the poloxamer gel. A cubic liquid crystalline gel was formed by mixing glycerol monooleate with DPK-060; cubosomes were then prepared by dispersing the cubic gel in poloxamer solution. In most experiments, DPK-060 dissolved in poloxamer gel was included as a reference group. The *in vitro*/*ex vivo*/*in vivo* antimicrobial efficacy of the formulations was characterized, and the release profile and skin irritancy were assessed. GMO, glycerol monooleate.

This study applies new formulation strategies including different classes of nanocarriers to formulate DPK-060 for topical delivery, and characterizes these different dose formulations in terms of preclinical *in vitro, ex vivo*, and *in vivo* antimicrobial activity (see Figure 1 for a schematic overview of the study).

## Materials and Methods

### Peptide and Antibiotics

DPK-060 peptide (GKHKNKGKKNGKHNGWKWWW; molecular weight 2.5 kDa, net charge +8.5 at pH 5.5, random coil secondary structure) was produced using Fmoc solid phase technology at Bachem AG, Bubendorf, Switzerland. The peptide was identified by electrospray ionization mass spectrometry and the purity was assessed by HPLC. The purity of the peptide used in all studies was 98.5%. Bactroban, 2% ointment (GlaxoSmithKline, Brent-ford, UK) was used as control antibiotics in *in vivo* studies.

### Formulations

DPK-060 was first associated with the nanocarriers, whereupon the DPK-060-loaded nanocarriers were dispersed into an *in situ* gelling poloxamer formulation (Table 1). The nanocarriers were characterized in terms of size (Z-average particle diameter) and polydispersity index by dynamic light scattering prior to use (Boge et al., 2016; Umerska et al., 2016b). The chemical stability of the peptide in all formulations investigated has been verified (assay by HPLC >95%).

**Table 1:** Formulation properties.

**Formulation**	**Poloxamer 407 (wt%)**	**Nanocarrier**	**Nanocarrier components**	**Particle size, nanocarrier (nm)**	**Peptide loading**
Poloxamer gel	17	–	–	–	–
LNCs in poloxamer gel	17	LNC	Lecithin, medium-chain triglycerides, macrogol 15 hydroxystearate	50–80	Adsorption to nanocarrier
ML-LNCs in poloxamer gel	17	ML-LNC	Monolaurin, medium-chain triglycerides, macrogol 15 hydroxystearate	30–45	Adsorption to nanocarrier
Cubosomes in poloxamer solution	3	Cubosomes	Glycerol monooleate	200–300	Loading during nanocarrier formation

#### Poloxamer Gel Formulation

Poloxamer 407 (Kolliphor® P407; kindly provided by BASF, Ludwigshafen, Germany) and methyl paraben (Fluka, Buchs, Switzerland) were dissolved in 20 mM acetate buffer pH 5.5 in an ice bath for 1–2 h until the polymer was completely dissolved (23 wt% poloxamer 407 and 0.03 wt% methyl paraben). For formulations containing DPK-060 without a nanocarrier, DPK-060 was first dissolved in acetate buffer and then added to the polymer solution while stirring. The final concentrations of poloxamer 407 and methyl paraben were 17 wt% and 0.02 wt%, respectively; the final concentrations of DPK-060 were 0.25 wt%, 0.5 wt%, and 1.0 wt%, corresponding to approximately 1.0 mM, 2.0 mM, and 4.0 mM, respectively.

#### Formulations With Lipid Nanocapsules

Lipid nanocapsules (LNCs) were prepared as described previously (Valcourt et al., 2016; Umerska et al., 2017). LNCs were composed of lecithin (Lipoid® S75–3; kindly provided by Lipoïd Gmbh, Ludwigshafen, Germany), medium chain triglycerides (Labrafac^®^ WL1349; kindly provided by Gattefossé S.A., Saint-Priest, France), and macrogol 15 hydroxystearate (Kolliphor® HS 15; BASF, Ludwigshafen, Germany). Briefly, lecithin (75 mg), triglycerides (846 mg), macrogol 15 hydroxystearate (1,028 mg), sodium chloride (90 mg), and 20 mM acetate buffer pH 5.5 (3 ml) were mixed and homogenized under magnetic stirring. The resulting emulsion was then heated to 90°C and cooled to 60°C 2 times, and then heated to 90°C and cooled to 78°C, to obtain reversible emulsion phase inversions. After the temperature of 78°C (the phase inversion temperature) had been reached, an irreversible shock was induced by a rapid dilution with 12.5 ml 4°C purified water to reach room temperature. For monolaurin (ML)-LNCs, lecithin was replaced by monolaurin (Sigma Aldrich, Lyon, France); monolaurin (300 mg), triglycerides (930 mg), macrogol 15 hydroxystearate (770 mg), sodium chloride (90 mg), and 20 mM acetate buffer pH 5.5 (3 ml) were mixed. The resulting emulsion was then heated to 60°C and cooled to 20°C 2 times, and then heated to 60°C and cooled to 37°C. After the temperature of 37°C (the phase inversion temperature) had been reached, the dispersion was rapidly diluted with 20°C purified water to a volume of 10 ml. The particle size of the LNCs and ML-LNCs was in the range of 50–80 nm and 30–45 nm, respectively, with a polydispersity index < 0.1.

To prepare peptide-loaded LNCs/ML-LNCs, the LNCs/ML-LNC dispersions were mixed with 160 mg/g (16 wt%) DPK-060 in acetate buffer at a ratio of 3:1 LNCs/ML-LNCs:DPK-060 solution, for 2–3 h [sufficient for peptide molecules to achieve a dynamic equilibrium between the LNCs and the surrounding medium (Umerska et al., 2016b)], resulting in a peptide concentration of 40 mg/g (4 wt%).

Finally, the DPK-060-loaded LNCs/ML-LNCs were added to the poloxamer gel in a 1:3 ratio while stirring at slow speed (300–500 rpm) in an ice bath for 60 min, which resulted in a homogenous viscous liquid. The final concentration of poloxamer 407 was 17 wt%; the final concentrations of DPK-060 were 0.25 wt%, 0.5 wt%, and 1.0 wt%, corresponding to approximately 1.0 mM, 2.0 mM, and 4.0 mM, respectively. The final concentrations of LNCs/ML-LNCs were five-fold higher than the DPK-060-concentrations, i.e., 1.25 wt%, 2.5 wt%, and 5.0 wt%.

The adsorption efficiencies of DPK-060 in LNCs and ML-LNCs were 33.8 ± 3.5% and 33 ± 5%–42 ± 2%, respectively, (manuscript by Matougui et al., in preparation; Rozenbaum et al., 2019).

#### Formulations With Cubosomes

Liquid crystalline nanoparticles (LCNPs) with cubic phase structure, i.e., cubosomes, were prepared as described previously (Boge et al., 2016). In brief, molten glycerol monooleate (Capmul-90 EP/NF; kindly provided by Abitec Corp., Columbus, OH) at 50°C was mixed with DPK-060 (at 28, 56, or 111 mg/ml) in acetate buffer (20 mM pH 5.5) at 22°C to reach a final concentration of 70:30 wt% glycerol monooleate:buffer with DPK-060 and was then allowed to equilibrate for 2 days at 22°C to form a cubic gel. Typical weights of prepared liquid crystalline gels were 1.7 g. Cubosomes were formed by first dispersing 1.5 g liquid crystalline gel in 3.5 g 20 mM acetate buffer pH 5.5 containing 3 wt% poloxamer 407 for 1 min using a Ultra-Turrax high-shear mixer (IKA T25, Staufen, Germany) with a diameter of 8 mm at 15,000 rpm, followed by sonication using a Vibracell Probe Sonicator (Sonics and Materials, Inc., Newtown, CT) with a 6 mm probe operating in pulse mode (3 s sonication followed by 7 s break) over a period of 10 min. This resulted in cubosomes in the size range of 200–300 nm with a polydispersity index about 0.3. The preparation of cubosomes using the shearing and sonication was shown not to affect the chemical stability of DPK-060 as investigated by HPLC. The cubic structures of the particles were verified by small angle x-ray scattering (Boge et al., 2016). The final concentration of poloxamer 407 was 3% (higher concentrations influence the phase properties of the LCNPs); the final concentrations of DPK-060 were 0.25, 0.5 and 1.0 wt% (corresponding to approximately 1.0, 2.0, and 4.0 mM, respectively); the final concentration of cubosomes was 30 wt% in all formulations. Methyl paraben (0.02 wt%) was added to the final dispersions.

Loading of DPK-060 into the cubic liquid crystalline gel followed by dispersion, as was used in this study, has been previously shown to result in a high peptide entrapment of 50–70%, whereas post-loading of cubosomes results in only 10–20% of DPK-060 being associated with the particles at the condition of low ionic strength (Boge et al., 2016, 2017). Therefore, the method of DPK-060 loading into the cubic liquid crystalline gel, which results in highest peptide encapsulation efficiency, was selected in this study.

### Assessment of Antimicrobial Activity *in vitro*

#### Minimum Microbicidal Concentration Assay

The microbicidal effect of DPK-060 in different formulations was assessed against *Staphylococcus aureus* (ATCC 29213; American Type Culture Collection, Manassas, VA) using a minimum microbicidal concentration (MMC) assay as described (Björn et al., 2016). The MMC assay was performed in 100 × diluted brain-heart infusion broth (BHI_dil_; Håversen et al., 2000, 2010; Kondori et al., 2011). The minimal peptide concentration causing >99.6% reduction of microorganisms was defined as the MMC.

#### Time-Kill Assay

Time-kill assay against *S. aureus* (ATCC 29213) was performed as previously described (Boge et al., 2017) incubating 20 μl of a bacterial suspension (~1.8 × 10^7^ CFU/ml) with 0.25 ml of each test item and 1.48 ml of BHI_dil_.

### *In vitro* Release

Release of DPK-060 from the different formulations was monitored through dialysis as described previously (Boge et al., 2017) using a Float-A-Lyzer® G2 dialysis device with 100 kDa molecular weight cut-off (Spectrum Laboratories Inc., Rancho Dominques, CA). The dialysis experiments were performed at 0, 7, and 14 days of storage of the formulations at room temperature. The samples were allowed to dialyze in 20 mM acetate buffer pH 5.5 for 24 h at room temperature, whereupon a 450 μl aliquot was removed from the receptor side/dialysate and diluted with 50 μl formic acid. The concentration of DPK-060 was then analyzed in duplicate samples by UHPLC using gradient elution and UV detection at 214 nm (Boge et al., 2016). The samples were analyzed on an Acquity UHPLC system (Waters corp, Milford, MA). The injection volume was 5 μl and the sampler was kept at 5°C. The mobile phases were; A: acetonitrile and purified water with 0.1% TFA and 50 mM NaCl 5/95 and B: acetonitrile and purified water with 0.1% TFA and 50 mM NaCl 70/30. The separation was performed by gradient elution starting from 26% phase B (kept constant for 1.5 min) increased to 35% phase B in 11 min (kept constant for 3 min), followed by a wash step at 100% phase B (1.1 min) and an equilibration step at 26% phase B (3.4 min). The flow rate was 0.25 ml/min and the column was kept at 55°C. DPK-060 was detected at 214 nm and quantified by peak area, quantitative dilutions, and external calibration in the range of 0.01–0.25 mg/ml.

The release of DPK-060 from the poloxamer gel formulation with and without LNCs was also investigated by Franz diffusion cell studies. Aluminum oxide membranes (Anodisc, 25 mm, pore size 0.02 μm, Whatman, GE Healthcare, Buckinghamshire, UK) were mounted in Franz diffusion cells (PermeGear Inc., Hellertown, PA) with a 0.64 mm^2^ surface area and receptor volume of 5 ml. 10 mM phosphate buffer pH 7.4, 0.8% NaCl was used as receptor medium and allowed to equilibrate at 32°C for 30 min prior to the application of 100 μl formulation to the donor compartment. Aliquots of 200 μl were collected over the period of 36 h and analyzed by UHPLC as described above.

### *In vitro* EpiDerm Skin Irritation Test

The effect of DPK-060 in different formulations on cell viability was determined in compliance with OECD Test Guideline 439 by using the EpiDerm Skin Irritation Test (EPI-200-SIT; MatTek In Vitro Life Science Laboratories, Bratislava, Slovakia) according to manufacturer's instruction.

### *Ex vivo* Wound Infection Model Using Pig Skin

The ability of DPK-060 in different formulations to reduce colonization of *S. aureus* (ATCC 29213) was evaluated in an *ex vivo* wound infection model using pig skin as previously described (Björn et al., 2016).

### Mouse Model of Surgical Site Infection

The injury, infection with *S. aureus* (ATCC 29213), and sample collection/analysis for surgical site infection model were carried out in female mice of CD1 strain as previously described (Håkansson et al., 2014). *In vivo* experiments were performed after prior approval from the local Ethics Committee for Animal Studies at the Administrative Court of Appeals in Gothenburg, Sweden (approval number: Dnr 26-2015).

The treatment-related systemic toxicity was assessed by observing general behavior and clinical signs including body posture, central excitation, mood, and motor activity. The local tolerability was assessed by observing incidence of erythema and oedema.

The doses of DPK-060 used in *ex vivo* and *in vivo* studies refer to the total concentrations of DPK-060 in the formulation and do not take into consideration the potential differences in the release pattern.

### Statistical Analysis

Statistical significance between the groups was calculated using one-way ANOVA followed by LSD *post-hoc* test, with a value of *P* <0.05 considered statistically significant. The statistical analyses were performed using SPSS Statistics (v24; IBM Corporation, Armonk, NY).

## Results

### *In vitro* Antimicrobial Potency

The MMC for DPK-060 in acetate buffer or when formulated with different nanocarriers in poloxamer gel was in the range of 1–5 μg/ml (Table 2). No antibacterial activity was detected when these formulations were tested without any DPK-060 added (data not shown).

**Table 2:** MMC for DPK-060 in different formulations against *S. aureus*.

**Test item**	**MMC (μg/ml)**
DPK-060 in acetate buffer	4.9
DPK-060 in poloxamer gel	1.2–2.4
DPK-060-loaded LNCs in poloxamer gel	2.4
DPK-060-loaded ML-LNCs in poloxamer gel	1.2
DPK-060-loaded cubosomes in poloxamer solution	4.9

*The MMC test is a semiquantitative assay; the results are presented either as one concentration value or, in case there was a variation between the repetitions, as a concentration range*.

**Figure 2:**
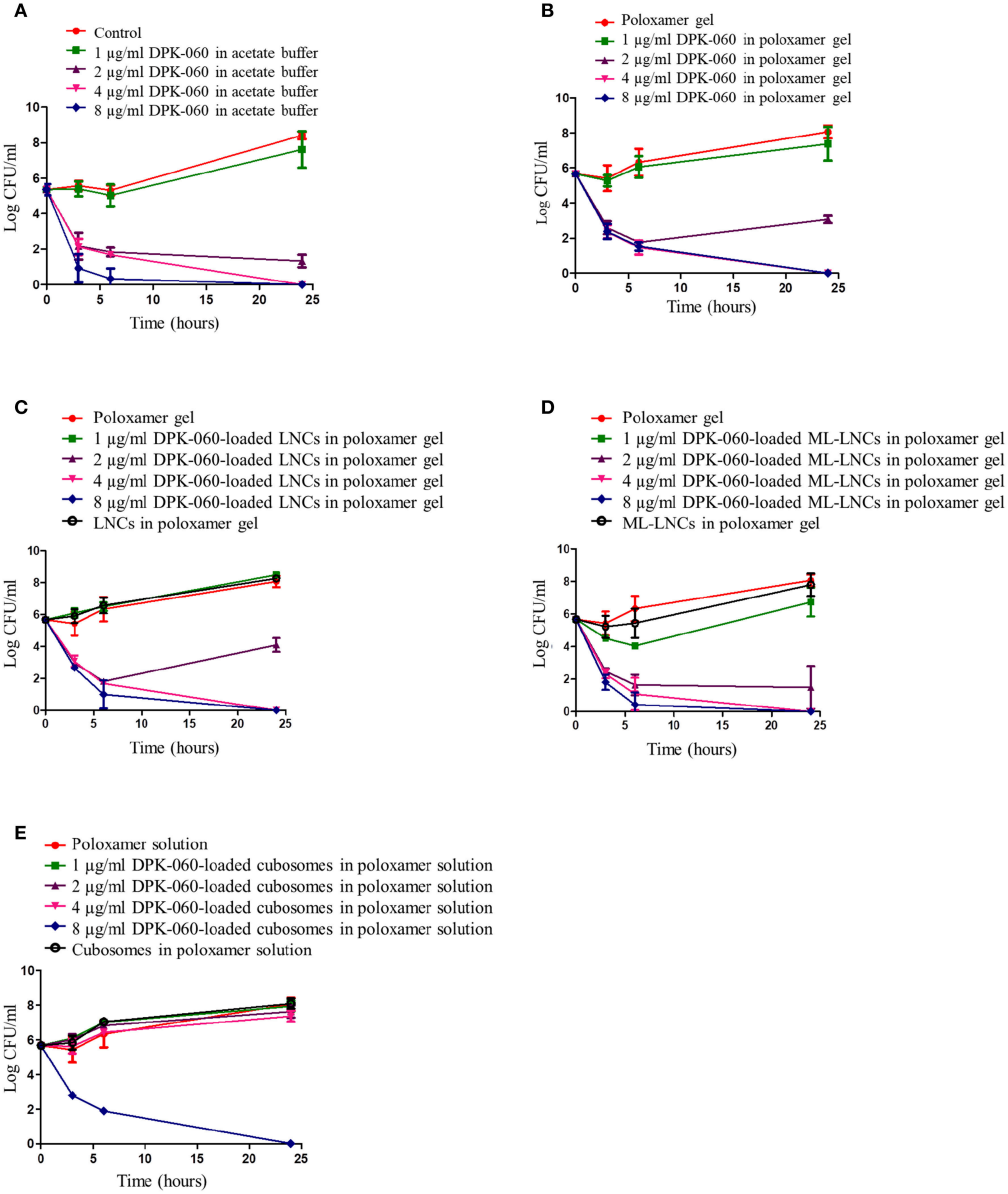
Time-kill assay for DPK-060 formulated in acetate buffer **(A)**, poloxamer gel **(B)**, or in different nanocarriers in poloxamer gel **(C–E)** against *S. aureus*. Data are mean ± SD (*n* = 3). Samples were considered as bactericidal if there was at least 3 log reduction in the CFU number compared with the starting inoculum.

DPK-060 in acetate buffer or in poloxamer gel displayed bactericidal effect in time-kill assay at concentrations equal or higher than 2 μg/ml with a significant reduction in the CFU numbers detected after 3 h of incubation (Figures 2A,B). DPK-060-loaded LNCs and ML-LNCs in poloxamer gel inhibited bacterial growth to the extent similar to that observed with DPK-060 in acetate buffer/poloxamer gel (Figures 2C,D). DPK-060-loaded cubosomes in poloxamer solution were less effective: the bactericidal effect was observed only at the highest concentration tested (8 μg/ml; Figure 2E). Nanocarrier gels without DPK-060 did not display any significant inhibition in bacterial growth (Figures 2C–E).

### *In vitro* Release

**Figure 3:**
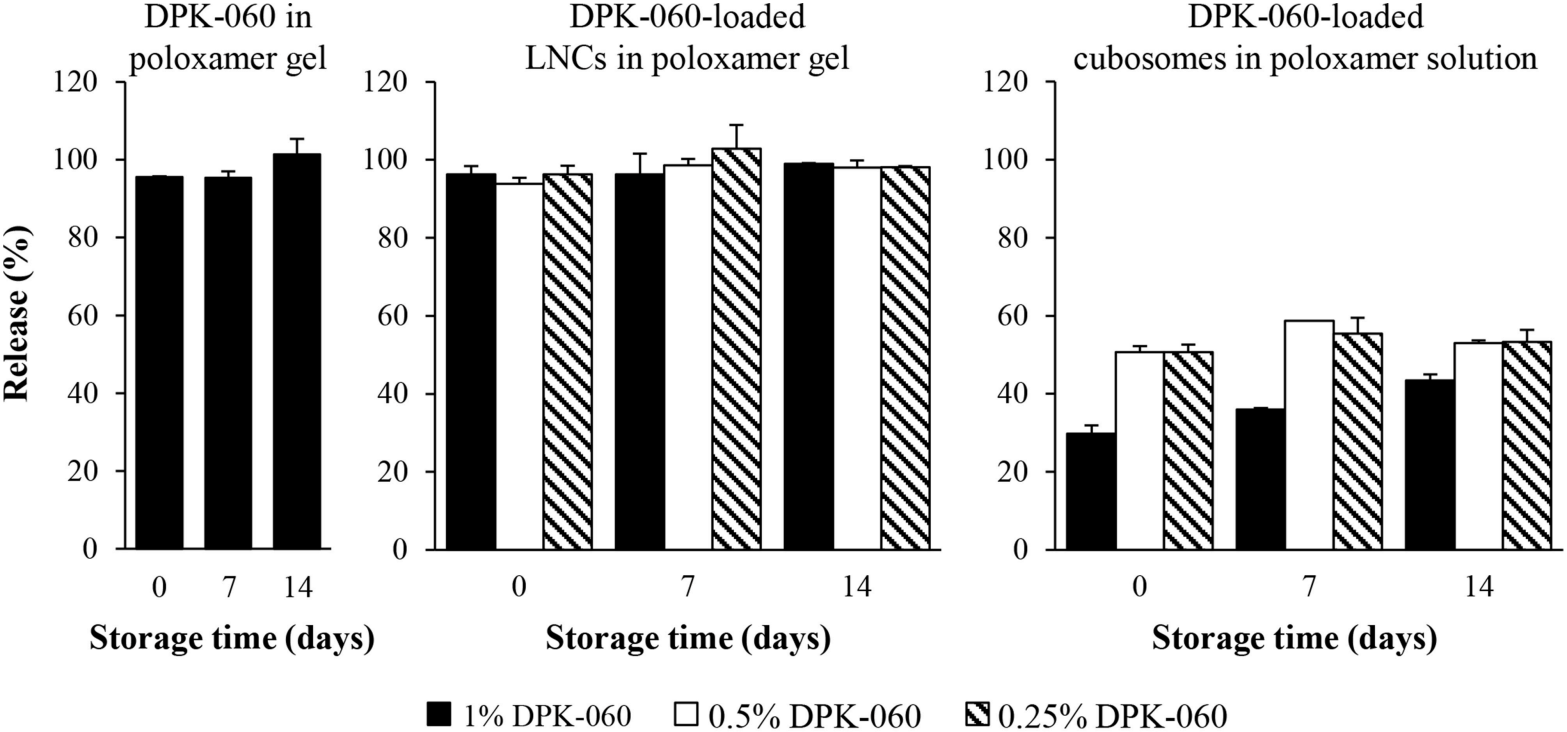
The release of DPK-060 from the formulations after different storage times (0, 7, or 14 days) investigated by dialysis method. Data are mean ± SD (*n* = 2).

The release of the peptide from DPK-060-loaded LNCs and cubosomes in poloxamer gel was first investigated by dialysis method. The results reveal that after 24 h of incubation, 100% of DPK-060 was released from the LNCs in poloxamer gel as well as from the poloxamer gel without any nanocarriers (Figure 3). In contrast, under these test conditions about 50–70% of DPK-060 remained encapsulated in the cubosomes (Figure 3). Of note, the release properties were similar comparing the formulations analyzed directly after preparation with those stored for 7 or 14 days before the dialysis experiment (Figure 3).

**Figure 4:**
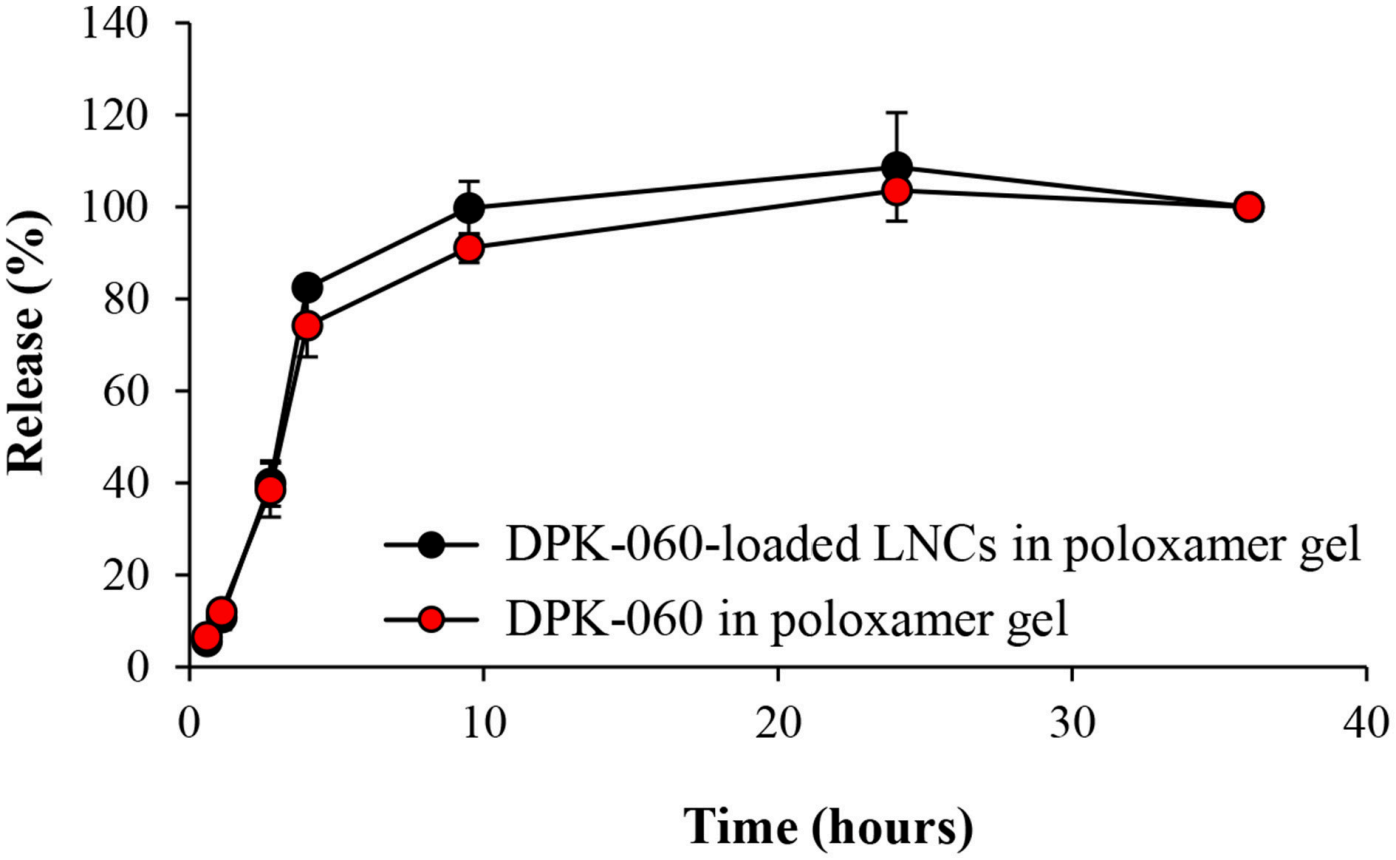
The release of DPK-060 investigated by Franz diffusion cell studies. Data are mean ± SD (*n* = 2).

The peptide release efficiency from DPK-060-loaded LNCs in poloxamer gel was further investigated by Franz diffusion cell studies. The results reveal identical release profile for DPK-060 in poloxamer gel with or without encapsulation of the peptide into LNCs: approximately 40% of DPK-060 was released within 3 h of incubation and full release (100%) was observed at 10 h (Figure 4).

### Antibacterial Effect in *ex vivo* and *in vivo* Wound Infection Models

**Figure 5:**
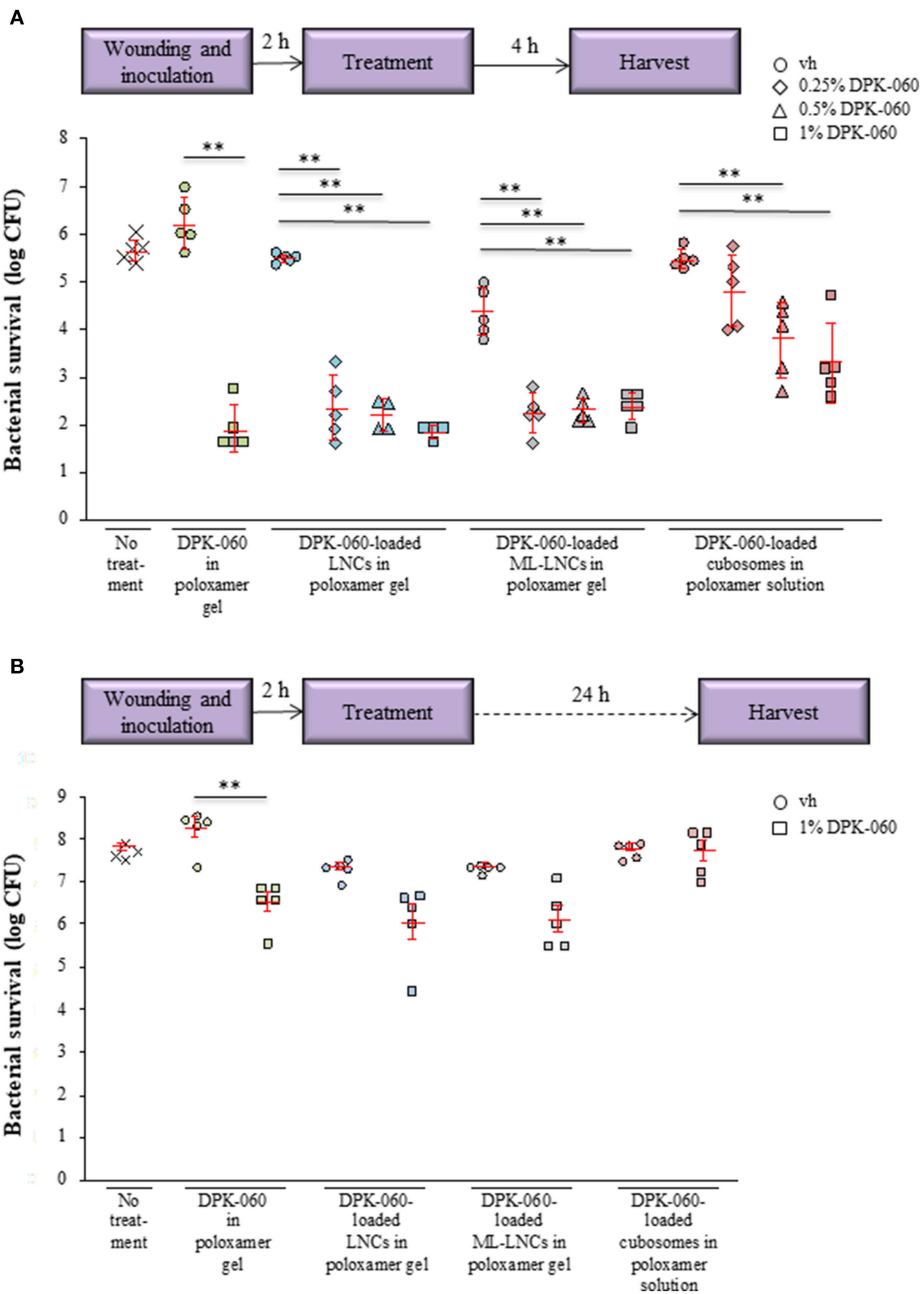
Antibacterial effect of DPK-060 formulated in poloxamer gel, or in different nanocarriers in poloxamer gel, in an *ex vivo* wound infection model using pig skin. The bacterial survival was assessed 4 h **(A)** or 24 h **(B)** after the treatment. Schematic presentation of the experimental setup is shown. Data on bacterial survival of *S. aureus* are mean ± SEM (*n* = 4–5 wounds per treatment group). ^**^ *P* < 0.01 (only the comparisons with the corresponding vehicle are shown). Vh, vehicle.

The antibacterial activity of DPK-060-loaded nanocarriers (0.25, 0.5, and 1.0% of DPK-060) was investigated in an *ex vivo* pig skin model. The formulations were administered 2 h post-infection with *S. aureus*, and the bacteria were harvested 4 h post-treatment (Figure 5A). DPK-060 in poloxamer gel, as well as DPK-060-loaded LNCs and ML-LNCs in poloxamer gel, significantly reduced the bacterial survival compared with sham (no treatment) as well as corresponding placebo (formulation only) and the concentration of 1% DPK-060 suppressed the bacterial survival with ≥99% vs. the sham (Figure 5A). The DPK-060-loaded cubosomes in poloxamer solution also diminished the microbial counts compared with sham/placebo; however, the anti-infectious potency was less pronounced compared with DPK-060-loaded LNCs/ML-LNCs (Figure 5A).

The *ex vivo* antimicrobial effect of selected formulations (1.0% DPK-060 in poloxamer gel, with or without nanocarriers) was further studied in the pig skin model after a longer follow-up period, i.e., the bacteria were harvested 24 h after the treatment (Figure 5B). In this setting, the bacterial survival was significantly reduced only in wounds treated with DPK-060 in poloxamer gel compared with corresponding placebo (Figure 5B).

**Figure 6:**
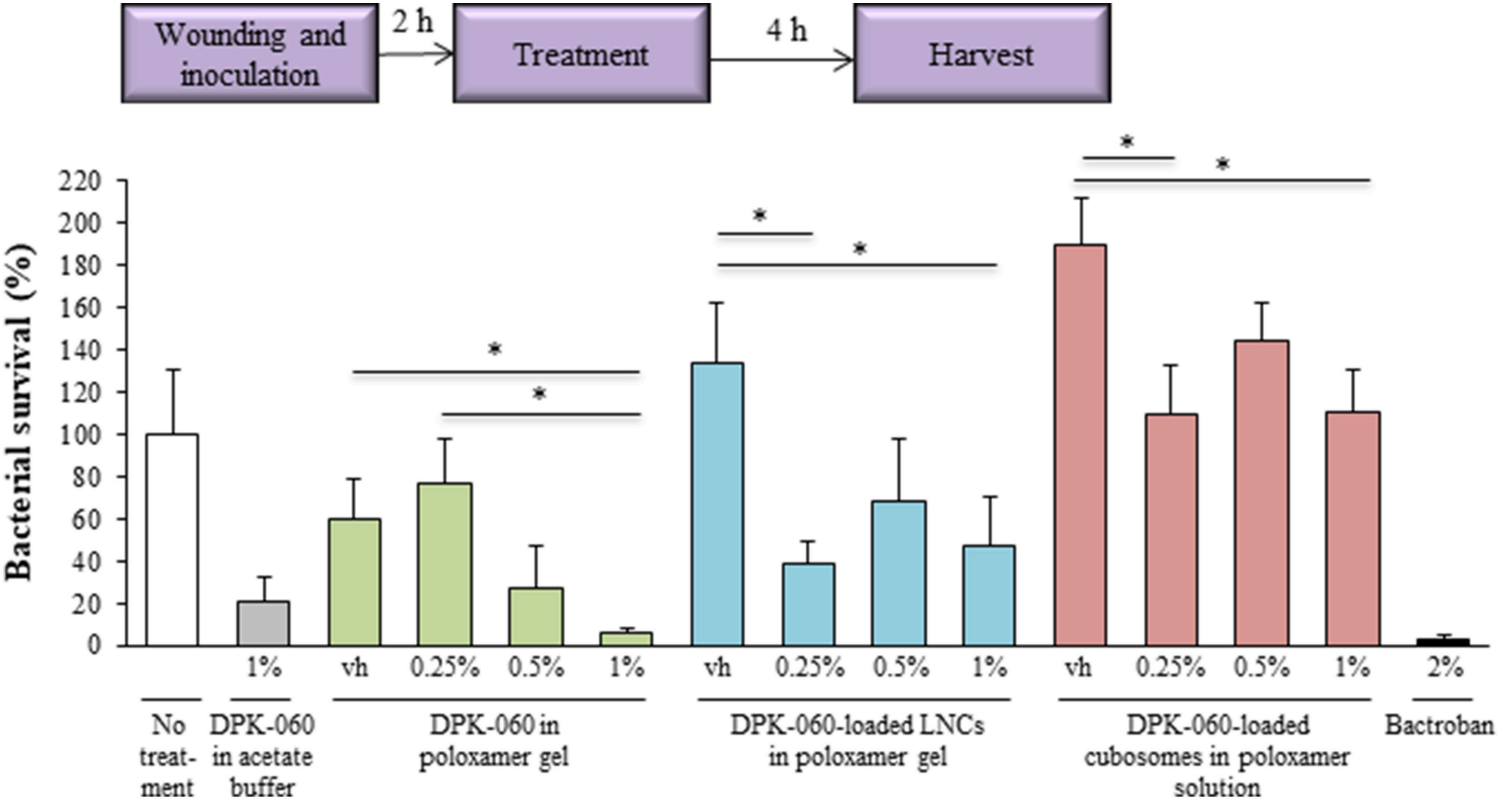
Antibacterial effect of DPK-060 formulated in poloxamer gel, or in different nanocarriers in poloxamer gel, in an *in vivo* mouse model of surgical site infection. Schematic presentation of the experimental setup is shown. Results are presented as average relative bacterial survival (%) of *S. aureus* compared to sham ± SEM (*n* = 5 mice per each treatment group). ^*^P < 0.05 (only the comparisons with the corresponding vehicle are shown). Vh, vehicle.

The antimicrobial effect of DPK-060-loaded nanocarriers (0.25, 0.5, and 1.0% of DPK-060) was also studied in a murine *in vivo* model of surgical site infection. In this model, a silk suture contaminated with *S. aureus* was implanted into an incision wound on the back of mice and assessment of the infection was performed 4 h post-treatment (Figure 6). The same model has been used previously to characterize the effect of systemic and topical antimicrobial agents and the results observed have closely correlated with efficacy in clinical trials with human subjects (Mcripley and Whitney, 1976; Gisby and Bryant, 2000; Rittenhouse et al., 2006). DPK-060 in poloxamer gel significantly suppressed the bacterial survival compared with sham as well as placebo (poloxamer gel) with a clear dose response relationship; the effect of 1% DPK-060 in poloxamer gel was comparable with the treatment with comparator 2% Bactroban (i.e., reduction in the bacterial survival with approximately 95% vs. the sham, Figure 6). Notably, DPK-060-loaded LNCs and cubosomes in poloxamer gel displayed minor/no antimicrobial effect in this model and no obvious dose response relationship was observed (Figure 6).

### Safety and Local Tolerability

In connection to the local application of the formulations in the surgical site infection model in mice, with or without DPK-060, no systemic toxicity or local tolerability concerns were visually observed in any of the mice. According to *in vitro* EpiDerm Skin Irritation Test, the test item is considered to display an irritating potential in case the viability is reduced by >50% of the values measured for negative control sample. All the formulations tested, with or without DPK-060, displayed the cell viability of ≥90% of the control sample.

## Discussion

The MMC results in this study corroborated the previous findings (Schmidtchen et al., 2009; Boge et al., 2016, 2017; Nordström et al., 2018) that human kininogen-derived peptide DPK-060 in acetate buffer or in poloxamer gel displays an *in vitro* bactericidal action against *S. aureus* with activities in the micromolar range. *In vitro*, different AMPs including DPK-060, when adsorbed to nanoparticles, have shown enhanced antimicrobial activity both against planktonic bacteria and in reducing biofilm growth (Umerska et al., 2016a, 2017; Boge et al., 2017, 2018). In specific, antimicrobial surfactant monolaurin used in ML-LNCs has been suggested to act in synergetic fashion with AMPs (Umerska et al., 2016a, 2017). In contrast, here we found that the MMC for DPK-060 formulated with different nanocarriers was in the similar range as the MMC for DPK-060 in acetate buffer or in poloxamer gel (1–5 μg/ml). Furthermore, in this study the DPK-060-loaded LNCs/ML-LNCs displayed similar microbicidal effect in time-kill assay compared with DPK-060 in acetate buffer/poloxamer gel (close-to-maximal effect at concentrations ≥2 μg/ml), whereas DPK-060-loaded cubosomes were less effective. In summary, while this study provides consistent evidence for the potent bactericidal action of DPK-060 *in vitro*, the encapsulation of this AMP into the nanocarriers failed to increase its antimicrobial efficacy in MMC or time-kill assay.

In addition to *in vitro* investigations, we also assessed the *ex vivo* and *in vivo* antimicrobial effect of DPK-060 in experimental wounds in pig skin and in mice, respectively, infected with *S. aureus*, which is one of the bacterial species most frequently causing human wound infections (Cardona and Wilson, 2015). We found that DPK-060 formulated in poloxamer gel caused a marked and statistically significant reduction in microbial counts with a clear dose-response relationship both *ex vivo* and *in vivo*. These data are consistent with the results of the previously completed clinical trial where total microbial count as well as count of Coagulase Negative Staphylococci (CoNS) and Gram-positive bacteria were significantly lower in eczematous lesions of patients with atopic dermatitis after 14 days of treatment with DPK-060 1% ointment (ClinicalTrials.gov Identifier: NCT01522391; EudraCT: 2007-007103-32).

Protection against proteolytic degradation by infection-affiliated enzymes has earlier been reported in *in vitro* studies when different AMPs were encapsulated into LNCs or cubosomes (Boge et al., 2017, 2019), which likely relates to the fact that the peptide is less accessible for proteolytic enzymes in nanoparticles compared with free peptide. Nanocarriers have also shown to enhance the skin penetration and delivery of several active substances (Lopes et al., 2006, 2007; Rattanapak et al., 2012; Seo et al., 2013). On the basis of these previous findings, we hypothesized that the efficacy of DPK-060 in treating wound infections would be enhanced *in vivo* when adsorbed to nanoparticles. In contrary, we found that the antibacterial effect of DPK-060 was diminished in a mouse model of surgical site infection when encapsulated into LNCs or cubosomes compared with formulating the peptide in poloxamer gel only. Notably, the results of *in vitro* release experiments revealed that more than 50% of DPK-060 remained encapsulated in the cubosomes after 24 h of incubation; in case only the non-encapsulated “free” peptide is giving rise to bacterial killing, this likely contributed to the low efficacy of DPK-060-loaded cubosomes. However, we found that the release profile for DPK-060 was similar in poloxamer gel with or without encapsulation of the peptide into LNCs (about 40% of DPK-060 was released within 3 h) and the reason for low efficacy of DPK-060-loaded LNCs *in vivo* remains elusive.

We did not observe any significant reduction in cell viability with DPK-060 in any nanocarrier formulation tested when assessed by *in vitro* EpiDerm Skin Irritation Test. Furthermore, we found no visible signs of systemic toxicity or local irritation in murine surgical site infection model in connection to the administration of DPK-060-containing formulations. This is consistent with the results of the clinical trial, where no serious adverse events (SAE) were reported after dermal application of DPK-060 in atopic dermatitis patients, whereas adverse events (AEs) were observed at similar frequency in the DPK-060 and placebo groups (ClinicalTrials.gov Identifier: NCT01522391).

In conclusion, the present study confirms that DPK-060 has the potential to be an effective and safe drug candidate for the topical treatment of microbial infections; however, under the applied test conditions, the adsorption of the peptide to nanocarriers failed to show any additional benefits. Notably, in this study, a single administration of the test formulations, combined with a short follow-up time, was used both in *ex vivo* and *in vivo* assessments, which is considered a limitation of the experimental design. Repeated application over several days, and a longer follow-up period, would closer resemble the clinically relevant situation and may lead to different results in terms of both efficacy and safety.

## Data Availability

The raw data supporting the conclusions of this manuscript will be made available by the authors, without undue reservation, to any qualified researcher.

## Author Contributions

JH: experimental planning and was responsible for *in vivo* and *in vitro* studies, scientific input, performed the *in vivo* experiments. LR: planning of studies, scientific input, responsible for the formulation studies. AU, JJ, TA, RR, and CB: performed *in vitro* studies and scientific input. LB and PT: performed formulation studies and scientific input. PKS: planning of *in vitro* and *in vivo* studies, scientific input. PS: planning of *in vitro* studies and scientific input. MM: overall responsible for the studies and corresponding author.

### Conflict of Interest Statement

At the time of the investigation, MM was employed at Promore Pharma AB and she continues to work for the company at consultancy basis. Promore Pharma is a biopharmaceutical company that develops peptide-based product candidates aimed for the bioactive wound care market. The remaining authors declare that the research was conducted in the absence of any commercial or financial relationships that could be construed as a potential conflict of interest.

## Abbreviations

AMP: antimicrobial peptide
LCNP: liquid crystalline nanoparticle
LNC, lipid nanocapsule: ML-LNC: monolaurin lipid nanocapsule
MMC: minimum microbicidal concentration.
